# A machine learning framework for interpreting phylogenetic tree patterns in interkingdom horizontal gene transfer

**DOI:** 10.3389/fbinf.2026.1891949

**Published:** 2026-09-21

**Authors:** Kevin Aguirre-Carvajal, Cristian R. Munteanu, Vinicio Armijos-Jaramillo

**Affiliations:** 1 Department of Computer Science and Information Technologies, Faculty of Computer Science, CITIC Research Center of Information and Communication Technologies, University of A Coruña, A Coruña, Spain; 2 Bio-Cheminformatics Research Group, Universidad de Las Américas, Quito, Ecuador; 3 Carrera de Ingeniería en Biotecnología, Facultad de Ingeniería y Ciencias Aplicadas, Universidad de Las Américas, Quito, Ecuador

**Keywords:** computational evolutionary biology, interkingdom horizontal gene transfer, machine learning, phylogenetic pattern classification, phylogenomics

## Abstract

**Introduction:**

Horizontal gene transfer (HGT), the movement of genetic material between unrelated organisms, is widely recognized as an important driver of genome evolution in bacteria. In eukaryotes, however, the evolutionary impact of HGT remains debated. The identification of interkingdom HGT (iHGT) is especially challenging due to the lack of gold standard methods. While automated approaches for iHGT candidate detection exist, the interpretation of phylogenetic tree topologies into biologically meaningful evolutionary patterns has traditionally depended on expert manual inspection, a process that is subjective, difficult to reproduce, and not scalable to large datasets.

**Methods:**

We present a computational framework that formalizes phylogenetic tree interpretation as a supervised machine-learning problem. We define five recurrent phylogenetic patterns (iHGT, NoHGT, Limited donor evidence, Multiple major clades, and Patchy phylogeny) and developed a feature-extraction pipeline that captures taxonomic composition and phylogenetic topology through six biologically interpretable descriptors derived from unrooted gene trees. Several machine-learning algorithms were evaluated using repeated stratified cross-validation, and model interpretability was assessed through permutation importance, SHAP analysis, rule-based baselines, and feature ablation. Performance was further validated on simulated datasets, real biological iHGT candidates, and an independently annotated external dataset.

**Results:**

A Random Forest (RF) classifier achieved the best performance (AUC–ROC = 0.98; accuracy = 0.89). Topological distance and lineage-distribution features were identified as the strongest contributors to classification performance. Feature ablation and rule-based baseline comparisons demonstrated that model performance cannot be explained solely by explicit annotation rules and benefits from combining clade-composition and topology-based information. The RF classifier showed low misclassification rates on simulated and real biological datasets (7.8% and 10.43%, respectively), showed substantial agreement (69.4%) with an independently annotated external dataset, and consistently outperformed AVP (Alienness vs. Predictor), Alien Index, and HGT Index across all evaluated metrics.

**Conclusion:**

These results support the use of machine learning for the automated, reproducible, and scalable classification of expert-defined phylogenetic patterns. The framework also highlights that some topologies commonly interpreted as evidence of iHGT may reflect alternative evolutionary processes, emphasizing the need for cautious and context-aware inference.

## 1 Introduction

Horizontal gene transfer (HGT) refers to the movement of genetic material between non-related organisms, bypassing vertical inheritance. In bacteria, HGT is widely recognized as a major driver of genetic innovation and diversification. In contrast, its prevalence and evolutionary impact in eukaryotes remain the subject of ongoing debate. Particularly contentious is the occurrence of interkingdom horizontal gene transfer (iHGT), which involves gene transfer between organisms from different biological kingdoms. Given the profound genetic divergence separating these lineages, such events would be expected to be rare or even negligible. Nevertheless, an increasing number of studies have reported putative cases of iHGT in eukaryotes (Katz, 2015; Cote-L’Heureux et al., 2022; Ma et al., 2022; Wu et al., 2024).

At the same time, recent analyses have suggested that many reported iHGT cases may be better explained by alternative evolutionary scenarios, and that methodological biases in detection approaches can substantially influence both the number and the nature of inferred candidates (Ku and Martin, 2016; Aguirre-Carvajal and Armijos-Jaramillo, 2025; Aguirre-Carvajal et al., 2025; Bremer et al., 2025).

In this context, standardized, replicable, and biologically grounded approaches are required to improve confidence in iHGT detection and to more accurately assess its evolutionary impact in eukaryotes. Two principal methodological frameworks are commonly employed to identify HGT candidates: parametric and phylogenetic approaches.

Parametric methods rely on genome-specific compositional signatures, such as GC content, third-codon position bias, codon usage, amino acid frequencies, and k-mer distributions, among others (Azad and Lawrence, 2012). These approaches have been applied predominantly in bacterial systems (Waack et al., 2006; Hudson et al., 2015; Soares et al., 2016; Narechania et al., 2025) and are based on detecting discrepancies between the compositional features of the putative donor fragment and those of the recipient genome. However, this strategy presents several limitations. Intrinsic heterogeneity within recipient genomes can lead to overestimation of HGT-derived segments. Moreover, transferred sequences undergo amelioration over time, progressively converging toward the compositional profile of the host genome. Consequently, parametric approaches have limited power to detect ancient or distantly related HGT events, like interkingdom lateral transferences.

Several tools implement this strategy, including SigHunter (Jaron et al., 2014), TF-IDF (Cong et al., 2016), IslandPath-DIMOB (Bertelli and Brinkman, 2018), HGT-DB (Garcia-Vallve et al., 2003), Islander (Hudson et al., 2015), and NECK (Narechania et al., 2025). More recently, synteny-based analyses have been incorporated to infer HGT events between closely related bacteria (Adato et al., 2015; Sevillya et al., 2020), with the aim of mitigating some of the compositional biases described above.

In contrast, phylogenetic approaches exploit the evolutionary signal contained in gene or protein sequences to identify incongruences with the expected species phylogeny. Such conflicts are typically detected through explicit phylogenetic reconstructions or through surrogate, implicit methods that estimate evolutionary distances or sequence similarity among genes (Ravenhall et al., 2015).

Implicit methods infer phylogenetic relationships without reconstructing explicit trees, enabling rapid screening for HGT candidates in large-scale datasets. These approaches typically rely on sequence similarity metrics and heuristic searches, which makes them computationally efficient. However, they can produce misleading inferences, as the most similar sequences identified through tools such as BLAST are not necessarily the closest evolutionary relatives in a robust phylogenetic framework (Koski and Golding, 2001).

Several tools have been developed based on this strategy, including DarkHorse (Podell and Gaasterland, 2007), HGTector (Zhu et al., 2014), HGTFinder (Nguyen et al., 2015), Alienness (Rancurel et al., 2017), BLAST2HGT (Li et al., 2018), and ShadowCaster (Sánchez-Soto et al., 2020).

Explicit phylogenetic methods can be broadly divided into two categories. The first involves direct inspection and analysis of reconstructed gene trees to infer HGT events or alternative evolutionary scenarios. This approach is commonly applied when researchers reconstruct phylogenies for candidates initially identified through implicit methods, or when investigating specific genes exhibiting unusual phylogenetic patterns. However, relatively few tools are available for the automated detection of HGT candidates through direct tree analysis; AvP (Koutsovoulos et al., 2022) is among the limited number of programs designed for this purpose.

The second category comprises tree reconciliation approaches, which compare gene phylogenies with a reference species tree. In principle, this framework has the capacity to discriminate among distinct evolutionary processes, including HGT, gene duplication and loss, and incomplete lineage sorting. Programs such as AnGST (David and Alm, 2011), HGTree (Choi et al., 2023), and RANGER-DTL (Bansal et al., 2018) implement this strategy. Nevertheless, analyses in fungi by Dupont and Cox (2017) indicate that reconciliation methods may exhibit limited sensitivity in distinguishing artifactual HGT candidates from vertically inherited genes. Although biologically coherent in theory, the broader application of these tools requires further validation under diverse evolutionary scenarios and, at present, may not be well suited for large-scale analyses such as whole-genome datasets. A further practical limitation is their dependence on rooted gene trees; automated rooting procedures are error-prone, and methods such as NOTUNG require rooted input, restricting applicability in high-throughput settings where reliable rooting cannot be guaranteed (Chen et al., 2000; Emms and Kelly, 2017).

Beyond the currently available methods for detecting HGT, most tools rely on implicit assumptions about how HGT events are expected to manifest in genomic data. However, many of these expected patterns may also arise from alternative evolutionary processes or from incomplete taxonomic sampling in public databases. For instance, implicit similarity-based approaches assume that a transferred gene should exhibit greater similarity to sequences from the putative donor lineage than to those from closely related species of the recipient lineage. Yet, this pattern may also result from the absence of unsampled or unsequenced homologs closely related to the recipient lineage.

Explicit phylogenetic approaches, in turn, often depend on the experience and interpretative criteria of the researcher when identifying incongruent topologies. This subjectivity can lead to either overestimation or underestimation of robust candidates, particularly in the context of iHGT events.

Given the methodological challenges associated with iHGT detection, we present PhyloHGT, a supervised machine learning framework for the systematic, reproducible classification of gene tree topologies as evidence of iHGT (https://github.com/kevynaguirre/PhyloHGT). The framework evaluates the global topology of each phylogenetic tree, considering the full distribution of major clades represented across all terminal nodes, and operates on unrooted gene trees, as none of the analyzed features depend on root placement. The user specifies a focal recipient major clade as input, which orients feature extraction. For each candidate tree, PhyloHGT assigns one of five expert-defined phylogenetic patterns (iHGT, NoHGT, Limited donor evidence, Multiple MC, and Patchy phylogeny), consolidated into three operational categories (iHGT, NoHGT, and Inconclusive); within the Inconclusive category, sub-pattern distinctions are retained because each reflects a different biological ambiguity and implies different follow-up strategies, providing actionable guidance rather than simply flagging candidates as uncertain. The framework is designed for two complementary uses: as a downstream classifier that systematically evaluates phylogenetic patterns in iHGT candidates flagged by other detection approaches, and as a standalone high-throughput screening tool to efficiently prioritize and filter large sets of pre-built gene trees, substantially reducing the burden of manual inspection that remains necessary in large-scale analyses (Li et al., 2022).

## 2 Methods

### 2.1 Conceptual framework for phylogenetic pattern classification

This study adopts the phylogenetic pattern classification framework introduced by Aguirre-Carvajal and Armijos-Jaramillo (2026), in which five recurrent topological configurations associated with interkingdom horizontal gene transfer (iHGT) were identified through expert inspection of large collections of atypical gene trees: iHGT, Limited donor evidence, Multiple major clades (MC), Patchy phylogeny, and NoHGT. Each pattern represents a distinct evolutionary scenario identifiable through gene tree topology. The goal of the present study is to formalize the classification of these patterns as a supervised machine-learning problem, replacing expert manual inspection with a reproducible and scalable computational framework.

Throughout this work, the terms 'recipient' and 'donor' are used as operational labels: the recipient refers to the user-specified clade of interest, the lineage being investigated for gene acquisition, and the putative donor refers to the most abundant other MC identified in the tree by the framework. These labels describe tree composition and do not constitute a claim about the biological direction of gene transfer.

To facilitate the interpretation of phylogenetic tree topology and the identification of recurrent phylogenetic patterns, taxa present in each tree were grouped into broader taxonomical categories referred to as major clades (MCs) (Table 1). In total, organisms were classified into 8 MCs, each encompassing multiple associated minor clades. These groupings are based on broad taxonomic categories derived from the NCBI Taxonomy database and previous studies (Katz, 2015; Aguirre-Carvajal and Armijos-Jaramillo, 2025). This hierarchical classification provides a standardized framework for summarizing gene tree taxonomic composition and facilitating comparisons among distantly related lineages.

**TABLE 1:** Classification scheme of major clades and associated minor clades applied in this study.

Major clade	Minor clades
Opisthokonta	Aphelida, Choanoflagellata, Filasterea, fungi, Ichthyosporea, Metazoa, Opisthokonta incertae sedis, Rotosphaerida, unclassified Opisthokonta
Sar	Alveolata, Rhizaria, Stramenopiles
Amoebozoa	Discosea, Evosea, Tubulinea, Amoebozoa incertae sedis, unclassified Amoebozoa
Archaeplastida	Viridiplantae, Rhodophyta, Glaucocystophyceae
Excavata	Metamonada, Malawimonadida, Discoba
Prokaryota	Bacteria, Archaea
Viruses	Viruses
Unclassified	Ancyromonadida, Apusozoa, Breviatea, Cryptophyceae, Haptista, Hemimastigophora, Provora, Rhodelphea, unclassified eukaryotes, CRuMs

Major clades represent broad taxonomic groups used to summarize the phylogenetic composition of each gene tree. Minor clades correspond to taxonomic lineages nested within these broader groups according to the NCBI, Taxonomy Browser (accessed 21 January 2026). Prokaryotes and viruses were treated as independent major clades to allow detection of potential transfer events involving these lineages.

Among the MCs, six correspond to major eukaryotic lineages. One of these categories (*Unclassified*) groups eukaryotic taxa that cannot be confidently assigned to a defined clade. In addition, prokaryotes and viruses were treated as independent MCs to allow detection of transfer scenarios involving these lineages when present in phylogeny.

Phylogenetic patterns were defined based on gene tree topology and the distribution of sequences across MCs, with each pattern representing a distinct evolutionary scenario. To illustrate iHGT, NoHGT, Limited donor evidence, Multiple MC and Patchy phylogeny patterns, conceptual phylogenetic topologies are shown in Figure 1.

**FIGURE 1:**
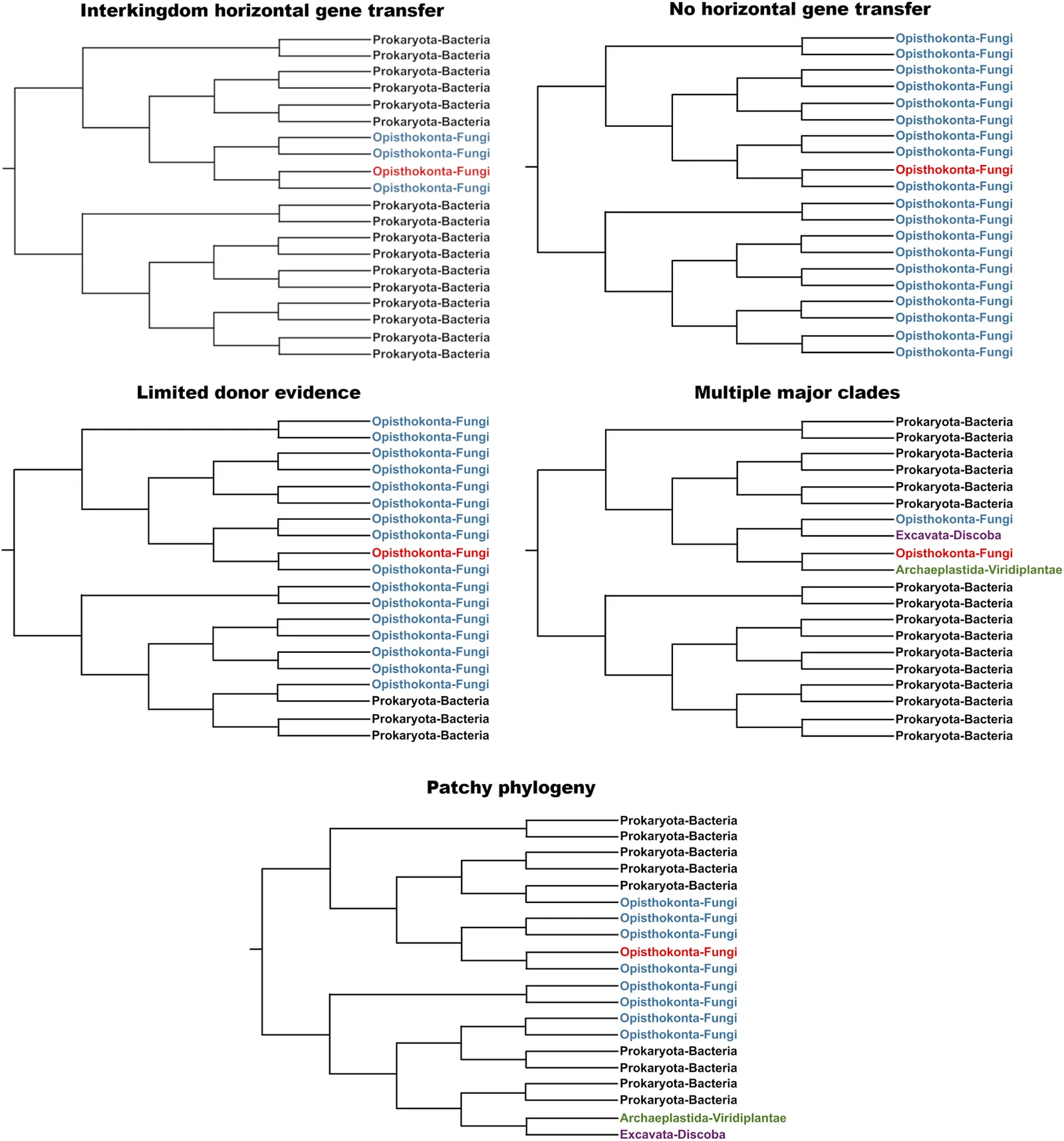
Schematic gene trees examples illustrate five recurrent evolutionary scenarios: interkingdom horizontal gene transfer (iHGT), No interkingdom horizonal gene transfer (NoHGT), Limited donor evidence, Multiple major clades (Multiple MC), and Patchy phylogeny. Trees are based on a representative example of gene transfer from bacteria (Prokaryota) to fungi (Opisthokonta). Colored labels indicate major clades, and the query sequence is highlighted in red. These conceptual topologies are intended to illustrate the distinguishing features of each phylogenetic pattern.

The iHGT scenario represents gene flow occurring exclusively between two major clades. This pattern is characterized by phylogenetic trees containing sequences from only 2 MCs: a putative donor and a recipient. In such trees, the query sequence belongs to the recipient MC and forms a monophyletic group nested within, or positioned as sister to, sequences from the donor MC. This topological configuration is consistent with the acquisition of the gene through an iHGT event and difficult to explain by alternative hypothesis.

The NoHGT class represents phylogenetic trees in which sequences belong to a single MC. In these cases, the tree contains query homologs sequences exclusively from that clade, with no representatives from other MCs. Because no cross-clade phylogenetic relationships are observed, the topology does not provide evidence supporting gene acquisition from another MC. This configuration is therefore interpreted as consistent with vertical inheritance within the lineage rather than iHGT. The NoHGT label refers specifically to the absence of phylogenetic evidence for iHGT into the user-specified clade of interest and does not exclude the possibility of HGT events occurring within that clade or gene transfers in other directions.

Limited donor evidence, Multiple MC, and Patchy phylogeny correspond to cases in which the phylogenetic signal is insufficient to confidently determine whether a gene candidate originated through iHGT, therefore they are grouped as Inconclusive patterns. In the Limited donor evidence pattern, phylogenetic trees contain sequences from 2 MCs, but the putative donor clade is represented by relatively few sequences compared with the recipient clade. This limited representation may indicate that the putative donor lineage is underrepresented in current databases, or that the direction of gene flow differs from the initial hypothesis. Because the scarcity of donor sequences reduces phylogenetic resolution and weakens confidence in inferring the direction of transfer, these cases do not provide sufficient evidence to robustly support an iHGT scenario. It should be noted that this topology is consistent with gene transfer in either direction between the 2 MCs; the pattern is therefore classified as Inconclusive rather than definitive iHGT.

The Multiple MC and Patchy phylogeny patterns correspond to phylogenetic trees containing sequences from three or more MCs. In the Multiple MC pattern, the query sequence clusters within a monophyletic group including sequences from the donor, recipient, and additional major clades. This topology is consistent with vertical inheritance followed by differential gene loss across lineages. Alternatively, an iHGT hypothesis would require invoking an ancient transfer followed by multiple independent gene losses, making the evolutionary scenario difficult to resolve.

In contrast, the Patchy phylogeny pattern is characterized by additional major clades distributed across different parts of the tree rather than forming a monophyletic group with the query sequence. Such topological configurations may result from several evolutionary processes, including differential gene loss, incomplete lineage sorting, or multiple independent horizontal gene transfer events between kingdoms.

### 2.2 Feature extraction from phylogenetic trees

Gene tree interpretation for iHGT detection relies on two core properties: the taxonomic composition of sequence homologs and their phylogenetic relationships relative to the query sequence (Podell and Gaasterland, 2007; Zhu et al., 2014; Koutsovoulos et al., 2022; Aguirre-Carvajal and Armijos-Jaramillo, 2025). To formalize these criteria as a machine-learning framework, six quantitative features were extracted from each gene tree and organized into two groups (Figure 2). For this purpose, sequences were classified as belonging to the recipient MC, the putative donor MC, or an additional MC (any MC distinct from both donor and recipient).

**FIGURE 2:**
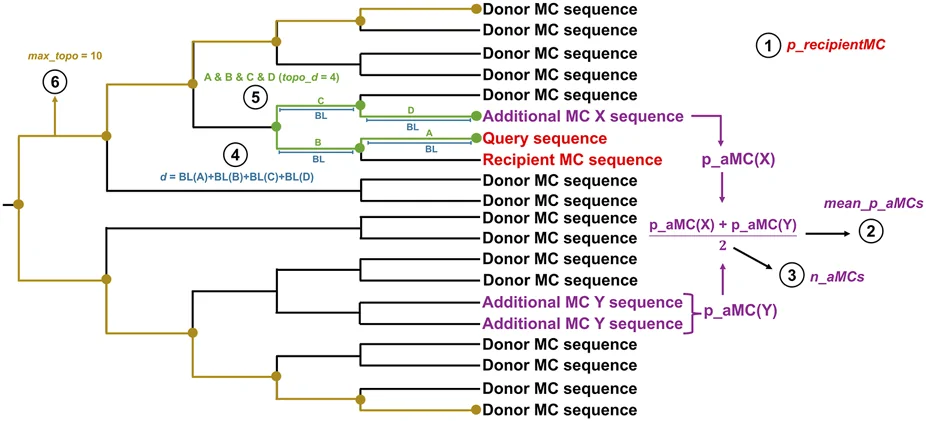
The schematic tree represents a hypothetical phylogeny containing sequences from the inferred donor major clade, recipient major clade, and additional major clades. The candidate (query) sequence and recipient major clade (MC) sequences are highlighted in red, whereas sequences belonging to the donor and additional clades are indicated in black, and purple, respectively. Numbered annotations represent the quantitative features extracted from the phylogenetic tree. (1) Proportion (p) of sequences belonging to the recipient MC (*p_recipientMC*). (2) Mean proportion of sequences belonging to additional (a) major clades (MCs) (*mean_p_aMCs*), calculated by first determining the proportion of terminal nodes for each additional MC (each distinct taxonomic group different from the donor and recipient MCs) and then averaging these proportions across all additional MCs present in the tree. (3) Number of additional major clades detected in the tree (*n_aMCs*). (4) Patristic distance (*d*) and (5) topological distance (*topo_d*) between the query sequence and the closest sequence from an additional major clade. Patristic distance corresponds to the sum of branch lengths (BL) along the path connecting two terminal nodes in the tree. In contrast, topological distance represents the number of edges (branches) separating the same pair of nodes, independent of branch length. (6) Maximum topological distance (*max_topo*) within the phylogeny. Together, these features capture both the taxonomic composition and the phylogenetic relationships that underpin the manual interpretation of gene tree topologies. Branch lengths are not drawn to scale, and the topology is shown for illustrative purposes.

The first group captures taxonomic composition through three features: the proportion of sequences belonging to the recipient MC (*p_recipientMC*; one in Figure 2), the mean proportion of sequences across additional MCs (*mean_p_aMCs*; two in Figure 2), and the total number of additional MCs detected in the tree (*n_aMCs*; three in Figure 2). The second group captures phylogenetic relationships between the query and sequences from additional MCs through three distance-based features: patristic distance to the closest additional MC sequence, normalized by tree patristic diameter (*d*; four in Figure 2), topological distance to the closest additional MC sequence (*topo_d*; five in Figure 2), and maximum topological distance within the tree (*max_topo*; six in Figure 2). When no additional MC sequences are present, *d, topo_d, mean_p_aMCs,* and *n_aMCs* are undefined and recorded as missing values, handled during model training.

All features were derived directly from phylogenetic trees using a fully reproducible pipeline implemented in Python v3.10.12. The pipeline accepts trees in NEXUS or Newick format with branch lengths in substitutions per site; a detailed description of the implementation, including taxonomic annotation, distance computation steps, and output format, is provided in Supplementary Material 1. The parameters used to run the pipeline are summarized in Supplementary Table S1.

### 2.3 Supervised model training and performance evaluation

Model training was conducted using a manually curated phylogenetic trees publicly available in the repository associated with the study of Aguirre-Carvajal and Armijos-Jaramillo (2025). The dataset comprises 1,012 phylogenetic trees, each represented by a set of six quantitative features extracted from gene tree topology and taxonomic composition. The model was trained to classify five phylogenetic patterns (iHGT, NoHGT, Limited donor evidence, Multiple MC, and Patchy phylogeny), which were later grouped into three broader categories (iHGT, NoHGT, and Inconclusive) for benchmarking comparisons. The distribution of phylogenetic patterns and their corresponding class assignments is summarized in Table 2. The dataset is accessible through Zenodo (https://doi.org/10.5281/zenodo.17552462).

**TABLE 2:** Distribution of phylogenetic patterns.

Grouped category	Phylogenetic pattern (training class)	Count
iHGT	iHGT	332
NoHGT	NoHGT	73
Inconclusive	Limited donor evidence	117
	Multiple MC	176
	Patchy phylogeny	314

Although the dataset is not balanced, the class imbalance ratio—defined as the ratio between the most represented class (iHGT) and the least represented class (NoHGT) at the phylogenetic pattern level—is approximately 4.5:1, which remains below levels commonly regarded as severe class imbalance in machine learning (e.g., ratios exceeding 10:1 or even 100:1) (Weiss, 2013; Leevy et al., 2018).

After completing the feature-extraction pipeline, seven supervised learning algorithms were evaluated to identify the model best suited for classifying the phylogenetic patterns defined above. The models evaluated included Random Forest (RF), Extreme Gradient Boosting (XGBoost), Multilayer Perceptron (MLP), Logistic Regression (LR), Gaussian Naïve Bayes (GNB), K-Nearest Neighbors (KNN), and Support Vector Classifier (SVC). All models were implemented in Python using the scikit-learn library v1.7.2 and XGBoost package v3.2.0.

Model-specific parameter settings are summarized in Table 3. Unless otherwise specified, all remaining parameters were set to their default values as implemented in the corresponding software versions used in this study. Minor adjustments were applied when required to ensure reproducibility or compatibility with multi-class classification.

**TABLE 3:** Machine learning models evaluated for phylogenetic pattern classification, including implementation details and parameter settings.

Model	Function	Parameters
RF	RandomForestClassifier ()	n_estimators = 100, random_state = 42
XGBoost	XGBClassifier ()	n_estimators = 100, eval_metric = “mlogloss”, random_state = 42
MLP	MLPClassifier ()	max_iter = 5000, hidden_layer_sizes=(100), random_state = 42
LR	LogisticRegression ()	solver = “lbfgs”, max_iter = 5000, random_state = 42
GNB	GaussianNB()	var_smoothing = 1e-09
KNN	KNeighborsClassifier ()	n_neighbors = 5
SVC	SVC()	kernel = 'rbf', probability = True, random_state = 42

Machine-learning models evaluated during the training phase, including their Python implementations and key parameter settings. Parameters not explicitly listed were kept at their default values as implemented in the corresponding software versions. Reported settings include parameters relevant to model configuration and reproducibility.

All models were trained using a standardized machine-learning pipeline implemented with the *Pipeline* class in scikit-learn. The pipeline consisted of two steps: (1) missing-value imputation using *SimpleImputer* with a constant strategy (*fill_value* = −1), and (2) the classification model under evaluation. Missing values arose when no homologs from additional major clades were present in the tree. This procedure ensured consistent preprocessing across all models.

To obtain robust and statistically comparable estimates of model performance, we implemented a repeated stratified k-fold cross-validation scheme using the *RepeatedStratifiedKFold* function. Specifically, five folds (*n_splits* = 5) were used, resulting in an 80% training set and a 20% test set for each split. The procedure was repeated 10 times (*n_repeats* = 10) with different random partitions (*random_state* = 42), producing 50 performance estimates per model while preserving class proportions across splits.

For each repetition, model performance was evaluated on the corresponding test partition using five metrics: area under the receiver operating characteristic curve (AUC–ROC), precision, recall, F1-score, and accuracy. For the multi-class setting, AUC–ROC was calculated using a one-vs-rest strategy. AUC-ROC, precision, recall, and F1-score were computed using weighted averaging to account for class imbalance, ensuring that each class contributed proportionally according to its frequency in the dataset. Accuracy was calculated as the proportion of correctly classified instances. These metrics capture discrimination ability (AUC–ROC), class-balanced predictive performance (precision, recall, F1-score), and overall accuracy. The resulting 50 performance estimates per model were used as input for statistical comparison using Friedman’s test followed by Nemenyi *post hoc* pairwise comparisons with Bonferroni correction (α = 0.05), implemented in R v4.5.3 using the packages stats v4.2.2 and PMCMRplus v1.9.12.

The model exhibiting the best performance in the preliminary comparison was therefore selected for hyperparameter optimization. Grid search and randomized search were performed using *GridSearchCV* and *RandomizedSearchCV* with the same repeated stratified cross-validation scheme described above. The optimized configurations were subsequently evaluated using the same statistical comparison framework applied in the initial model assessment.

### 2.4 Comprehensive model evaluation and interpretability

All analyses described in the following sections were performed using the best-performing model identified after model selection and hyperparameter optimization. To assess model performance comprehensively, class-specific ROC curves and AUC-ROC values were generated using a one-versus-rest strategy, and class-specific precision, recall, and F1-scores were calculated alongside the weighted metrics described above. Confusion matrices were computed per fold and summed across all cross-validation repetitions to characterize misclassification patterns. To investigate whether classification errors were associated with specific feature values, a follow-up examination was conducted by aggregating per-sample predictions across all cross-validation folds using majority voting, then comparing feature distributions between correctly classified and misclassified instances.

The influence of class imbalance was evaluated through two strategies: a class-weighted model trained using the class_weight = “balanced” parameter in scikit-learn, and a SMOTE-augmented model using the imbalanced-learn package with oversampling applied exclusively within training partitions to avoid information leakage.

Learning curve analysis was conducted using repeated stratified cross-validation (5-fold × 10 repeats) and balanced accuracy as the evaluation metric, across progressively larger training subsets ranging from 10% to 100% of available samples.

To assess whether the best-performing model outperforms a simpler interpretable alternative, a Decision Tree classifier was used as a rule-based baseline trained on the same six features. The optimal tree depth was selected through nested cross-validation using macro-F1 as the criterion (details in Supplementary Material 1); both classifiers were evaluated under the same outer cross-validation protocol (RepeatedStratifiedKFold, n_splits = 5, n_repeats = 10, random_state = 42). The decision rules learned on the full training dataset are provided in Supplementary Material 2. An ablation analysis was additionally performed by retraining the model on four reduced feature configurations: without *n_aMCs*, clade-composition only (*p_recipientMC, mean_p_aMCs, n_aMCs*), without proportion features, and distance-only (*topo_d, d, max_topo*); the rule-based baseline was included alongside all configurations for direct comparison.

To investigate individual feature contributions, permutation feature importance was calculated using the sklearn.inspection module, and SHAP analysis was performed using shap v0.49.1 to quantify feature contributions based on cooperative game theory principles.

For all analyses, 95% confidence intervals were estimated across the 50 validation folds using a t-distribution (mean ± t × standard error of the mean). Where multiple conditions were compared, statistical differences were assessed using a Friedman test with Nemenyi *post hoc* pairwise comparisons applied to macro-F1 scores (α = 0.05). Balanced accuracy and class-specific F1-scores were additionally computed for each evaluation and are provided in the supplementary tables referenced throughout the Results section.

### 2.5 Simulation of phylogenetic patterns

To validate model performance under controlled conditions, we simulated a total of 1,000 phylogenetic trees, comprising 200 replicates for each phylogenetic pattern described in Table 2. Simulations were conducted using HgtSIM v1.1.0 (Song et al., 2017) and restricted to single-celled organisms, as the framework targets microbial systems. The set of organisms included is provided in Supplementary Table S2.

Simulations modeled evolutionary scenarios in which bacterial organisms (Prokaryota) acted as donors, transferring genes into fungi (Opisthokonta). Ten widely conserved bacterial genes were selected as donor loci: murA, murB, murC, murD, murE, murF, lpxA, rpoD, fliC, and ddl (Opiyo et al., 2010; Shinde et al., 2021; Mayank, 2022; Xu et al., 2025; Xia et al., 2026). These genes were chosen as representative bacterial loci for simulation purposes, without implying exclusivity to prokaryotes. Reference protein sequences for each locus were identified via keyword searches in the NCBI Protein database and retrieved using Entrez Direct v24.0. Because HgtSIM operates on nucleotide sequences, amino acid sequences were back-translated into coding DNA using the EMBOSS backtranseq utility v6.6.0.0; direct retrieval of nucleotide records was avoided as preliminary tests resulted in reading frame inconsistencies. Recipient genome assemblies were obtained using the NCBI Datasets tool v18.14.0. A schematic overview of the simulation workflow and resulting phylogenetic patterns is provided in Figure 3.

**FIGURE 3:**
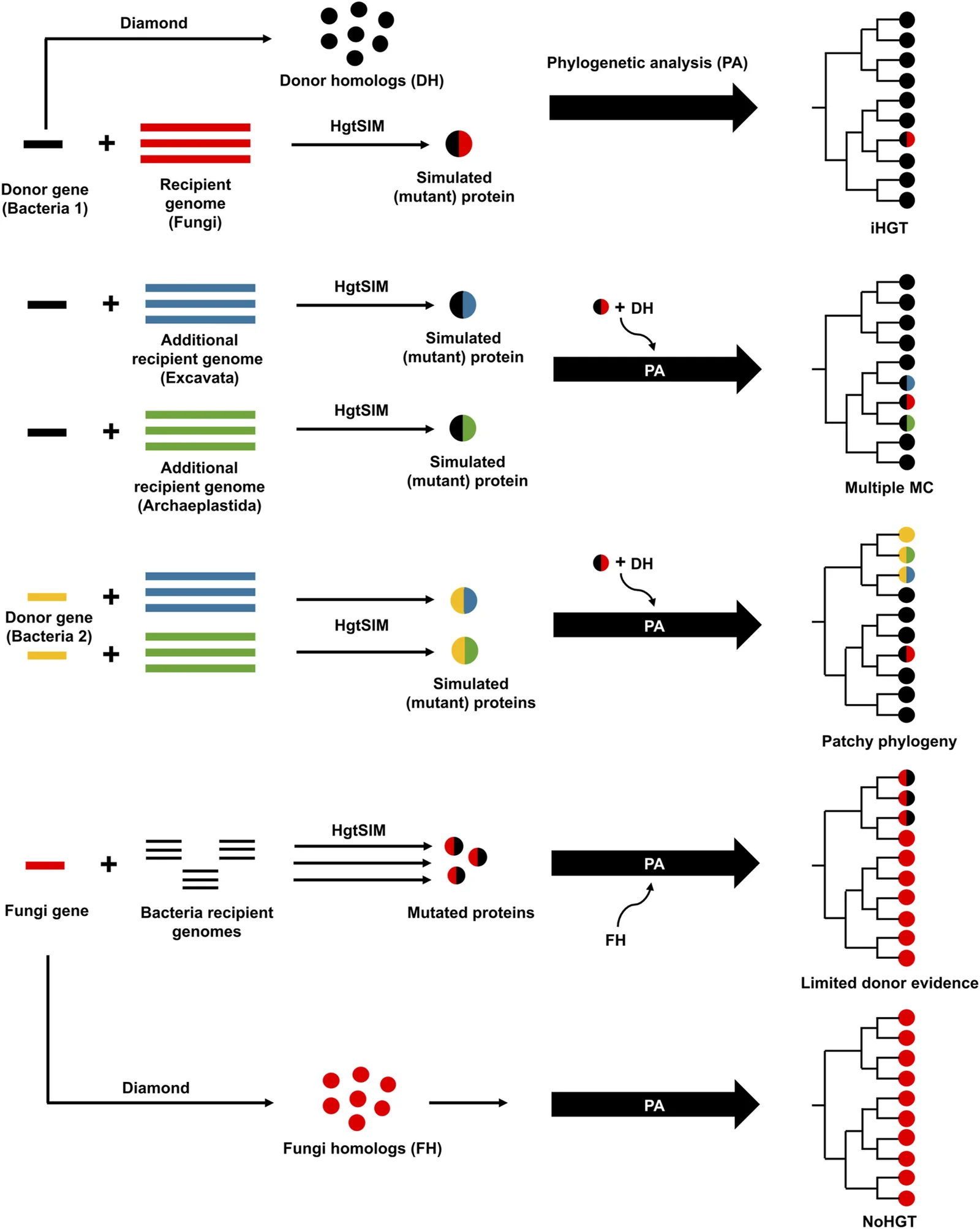
Schematic representation of how studied phylogenetic patterns were simulated using HgtSIM. Donor genes of prokaryotic origin (Bacteria one or Bacteria 2) are introduced into recipient fungal genomes (Opisthokonta) or other eukaryotic lineages to generate distinct evolutionary patterns. Homologous sequences are retrieved using DIAMOND, and phylogenetic analysis (PA) is performed to reconstruct gene trees. The resulting topologies correspond to five patterns: (i) interkingdom horizontal gene transfer (iHGT), where fungal sequences cluster within prokaryotic homologs; (ii) Multiple MC, where homologs are distributed across multiple major clades (MCs) but form a monophyletic group; (iii) Patchy phylogeny, characterized by discontinuous distribution of homologs across clades; (iv) Limited donor evidence, where donor sequences are sparsely represented; and (v) NoHGT, where no horizontal transfer signal is observed. Abbreviations: DH, donor homologs; FH, fungal homologs; PA, phylogenetic analysis.

As illustrated in Figure 3, scenarios were implemented as follows. Donor sequences were horizontally introduced into 20 distinct fungal genomes, generating 200 baseline simulated iHGT events. To generate the Multiple MC pattern, the transferred gene was subsequently introduced into taxa from MCs distinct from Opisthokonta, producing trees in which homologs are distributed across multiple major lineages while clustering in a monophyletic clade. To simulate the Patchy phylogeny pattern, the gene transferred into fungi originated from a single prokaryotic donor species, whereas homologs in other major clades were derived from different prokaryotic species independently carrying the same gene, generating discontinuous phylogenetic distributions. To simulate the Limited Donor Evidence pattern, 200 fungal genes were transferred into prokaryotic organisms, producing trees in which the user-specified clade of interest (Opisthokonta) is numerically dominant while the other MC (Prokaryota) is sparsely represented. To generate the NoHGT pattern, the same 200 fungal genes were retained without introducing any transfer event. Each simulation was assigned to a unique identifier to prevent duplication; simulation configurations and metadata are provided in Supplementary Table S3.

HgtSIM was executed using the default mutation ratio parameter and varying mutation intensity levels. Full usage instructions and parameter descriptions are available in the project documentation (https://github.com/songweizhi/HgtSIM). HgtSIM generates the mutated gene inserted into the recipient genome along with its translated protein sequence. Because the feature-extraction pipeline relies on NCBI protein accession identifiers to retrieve taxonomic metadata, and simulated sequences do not exist in the NCBI database, each simulated sequence was assigned the NCBI protein accession of the corresponding recipient species to enable taxonomic annotation during downstream phylogenetic analysis.

To obtain homologous sequences for phylogenetic analysis, protein similarity searches were performed using DIAMOND v2.1.9 against the NCBI NR database, using the transferred protein as query. For iHGT, Multiple MC, and Patchy phylogeny simulations, searches were restricted to prokaryotic sequences (--taxonlist 22,157; Bacteria and Archaea). For Limited donor evidence and NoHGT simulations, searches were restricted to fungal sequences (--taxonlist 4751). Hits were retained if they exhibited pairwise identity above 30%, query coverage above 60%, and E-value below 1 × 10^−5^. Retrieved sequences were combined with the simulated protein sequences into a single FASTA file.

For each simulation, homologous sequences were aligned using MAFFT v7.520 (Katoh and Standley, 2013) under automatic parameter selection. Ambiguously aligned regions were removed using Gblocks v0.91b with default parameters for protein sequences (Talavera and Castresana, 2007). The best-fit amino acid substitution model was selected using ModelTest-NG v0.1.7 (Darriba et al., 2020), and maximum-likelihood phylogenies were inferred with PhyML v3.3.20220408 (Guindon et al., 2010) with branch support evaluated using SH-like approximate likelihood ratio test values. Trees were exported in NEXUS format and annotated using a custom Python v3.10.12 script integrating taxonomic metadata from the corresponding GenBank records.

### 2.6 Generation of phylogenetic trees from published iHGT candidates

To evaluate model performance under real biological conditions, we compiled iHGT candidate genes from Aguirre-Carvajal and Armijos-Jaramillo (2026) and Aguirre-Carvajal et al. (2025). Prior to analysis, it was verified that none of these candidates had been previously used during model training. Redundant entries between the two studies were removed, resulting in a final dataset of 1,438 HGT candidates. Phylogenetic trees for the candidates reported by Aguirre-Carvajal and Armijos-Jaramillo (2026) were retrieved from the corresponding public repository (https://doi.org/10.5281/zenodo.18435322).

For candidates studied by Aguirre-Carvajal et al. (2025), phylogenetic trees were reconstructed. First, protein similarity searches were performed using DIAMOND, with the candidate protein used as the query against the same reference database described above. To ensure comprehensive taxonomic representation, two separate searches were conducted: one restricted to prokaryotic sequences (--taxonlist 22,157) and another restricted to eukaryotic sequences (--taxonlist 2759, corresponding to Eukaryota). The resulting hits were merged and filtered following the criteria described previously and subsequently processed through the phylogenetic reconstruction pipeline described in the previous section.

The inferred phylogenetic trees were manually inspected using Geneious Prime 2025.2.1 (https://www.geneious.com; accessed 26 February 2026) and classified according to the phylogenetic patterns defined in Table 2. This classification was performed exclusively through visual inspection of each tree’s topology and the taxonomic identity of its terminal nodes, prior to and independently of any quantitative feature computation. No numerical feature values were consulted during the labeling step, ensuring that the manual class assignments were not directly guided by the extracted model features. Phylogenetic trees from Aguirre-Carvajal and Armijos-Jaramillo (2026) were assigned the same phylogenetic patterns as reported in the original study.

### 2.7 Benchmark comparison of iHGT detection methods

Three methods were selected for benchmarking. AVP (Alienness vs. Predictor) v1.0.10 (Koutsovoulos et al., 2022) was chosen as the primary comparator because, like the proposed framework, it classifies candidates using phylogenetic tree topology; analyses were conducted in IQ-TREE mode and ingroup and exclusion group parameters are summarized in Supplementary Table S4. The Alien Index (Gladyshev et al., 2008) and HGT Index (Boschetti et al., 2012) were included as representatives of sequence-similarity-based approaches; both quantify the relative affinity of the query to donor versus recipient taxa from BLAST-based searches, with positive scores indicating stronger affinity to non-recipient taxa and negative scores assigned to NoHGT. Because the Alien Index and HGT Index produce inherently binary classifications, the Inconclusive category was merged with NoHGT for all methods to ensure a consistent evaluation framework. For detailed descriptions of each metric and their original formulations, readers are referred to the respective publications.

Phylogenetic trees from both simulated (1,000 trees) and real biological datasets (1,438 trees) were processed through the feature extraction pipeline and classified by the best-performing model (parameters in Supplementary Table S5). Performance of all four methods was evaluated using accuracy, precision, recall, F1-score, and Matthews Correlation Coefficient (MCC), computed using the confusionMatrix function (caret v6.0–93) and mcc function (mltools v0.3.5) in R.

### 2.8 External validation using an independently annotated dataset

To assess the external validity of the proposed framework, the best-performing model was evaluated on two independently annotated datasets comprising a combined total of 579 phylogenetic candidates. In Crisp et al. (2015), 73 bacterial-to-human HGT candidates were classified based on PCR experimental validation into three categories: HGT confirmed by PCR (iHGT), HGT not confirmed by PCR (NoHGT), and ambiguous (Inconclusive). In Ciach et al. (2024), 506 candidates were annotated through gene tree-species tree reconciliation into supported_hgt (iHGT), congruent clades with vertical transfer (NoHGT) and unsupported_hgt (Inconclusive) (Zenodo record 10649525).

For both datasets, candidates were matched to NCBI records, phylogenetic trees were reconstructed following the same workflow described in the preceding sections, and the six phylogenetic features were extracted and used as input for the best-performing classifier. The resulting classifications were compared with the original annotations using overall agreement as evaluation metric.

A global overview of the complete analytical workflow is provided in Figure 4. The complete implementation, including feature extraction, model training, and benchmarking scripts, is publicly available at https://github.com/kevynaguirre/PhyloHGT; phylogenetic trees from simulated and real biological datasets are deposited in the same repository.

**FIGURE 4:**
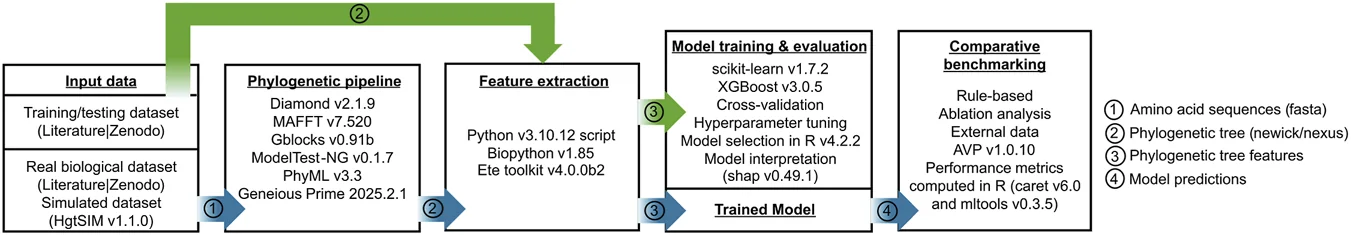
Computational workflow of the proposed framework for phylogenetic pattern classification and benchmarking. The workflow begins with three sources of input data: (i) a curated training/testing dataset, (ii) real biological datasets, and (iii) simulated datasets generated with HgtSIM. Protein sequences are processed through a phylogenetic reconstruction pipeline to generate gene trees. These trees are subsequently used for feature extraction, where taxonomic metadata and phylogenetic descriptors are computed using Python-based tools. The resulting feature matrices are used to train and evaluate machine-learning models, including cross-validation, hyperparameter tuning, model selection and interpretation. The best-performing model is further evaluated using rule-based analysis, ablation studies, and independent external datasets. Then, its classifications are benchmarked against AVP. Performance metrics are computed in R. Green arrows represent the training and model development workflow, whereas blue arrows indicate the benchmarking and evaluation workflow applied to independent datasets. Numbered arrows indicate data types transferred between steps: (1) amino acid sequences, (2) phylogenetic trees, (3) extracted features, and (4) model predictions.

## 3 Results

### 3.1 Models analysis

A feature-extraction pipeline was developed to derive quantitative descriptors from phylogenetic trees. The extracted features are provided in Supplementary Table S6 and were used to train multiple machine-learning models to classify phylogenetic patterns.

The predictive performance of all evaluated models is summarized in Table 4. The Random Forest (RF) model achieved the best performance across all evaluation metrics, with statistically significant advantages over several alternative models according to pairwise Nemenyi comparisons. XGBoost showed comparable performance for most metrics but exhibited slightly lower AUC-ROC. Logistic Regression and Gaussian Naive Bayes achieved intermediate performance, while MLP, KNN, and SVC performed progressively worse. Although RF and XGBoost were statistically indistinguishable for several individual metrics, RF was selected for further development based on its consistently highest mean values across all metrics and its greater interpretability through stable feature importance estimates.

**TABLE 4:** Performance comparison of machine-learning models trained to classify phylogenetic patterns.

Model	AUC-ROC	Precision	Recall	F1-score	Accuracy
RF	0.9806 ± 0.0051 (a)	0.8894 ± 0.019 (a)	0.8891 ± 0.0186 (a)	0.8878 ± 0.019 (a)	0.8891 ± 0.0186 (a)
XGBoost	0.979 ± 0.0058 (ab)	0.8854 ± 0.0198 (a)	0.8843 ± 0.0198 (a)	0.8836 ± 0.0198 (a)	0.8843 ± 0.0198 (a)
LR	0.9726 ± 0.0054 (bc)	0.8192 ± 0.0283 (b)	0.8154 ± 0.0208 (b)	0.8018 ± 0.0234 (b)	0.8154 ± 0.0208 (b)
GNB	0.9614 ± 0.0075 (cd)	0.7638 ± 0.0431 (bc)	0.7666 ± 0.0227 (bc)	0.744 ± 0.0232 (bc)	0.7666 ± 0.0227 (bc)
MLP	0.9257 ± 0.0224 (de)	0.6995 ± 0.0647 (cd)	0.6934 ± 0.0599 (cd)	0.6396 ± 0.0667 (cd)	0.6934 ± 0.0599 (cd)
KNN	0.7769 ± 0.0254 (ef)	0.5398 ± 0.0414 (de)	0.5508 ± 0.0333 (de)	0.53 ± 0.0329 (de)	0.5508 ± 0.0333 (de)
SVC	0.6376 ± 0.0178 (f)	0.2779 ± 0.0883 (e)	0.2954 ± 0.0267 (e)	0.2385 ± 0.0345 (e)	0.2954 ± 0.0267 (e)

Values represent the mean ± standard deviation calculated across repeated training–testing partitions. Different letters indicate statistically significant differences among models within each evaluation metric. The individual performance values obtained across repetitions are provided in Supplementary Table S7. Statistical significance was assessed using the Friedman test, followed by pairwise comparisons with the *post hoc* Nemenyi test; the corresponding p-values are reported in Supplementary Table S8. Models sharing the same letter were not significantly different, whereas different letters indicate statistically significant differences.

The RF model was further evaluated under two hyperparameter tuning strategies—randomized search and grid search—with optimal configurations provided in Supplementary Table S9. Neither strategy yielded meaningful improvements over the baseline model: grid search produced comparable performance across most metrics but performed significantly worse for AUC-ROC, while randomized search performed significantly worse for precision, recall, F1-score, and accuracy (Supplementary Table S8). The baseline RF model was therefore retained for subsequent implementation.

### 3.2 Comprehensive performance evaluation of the Random Forest classifier

Class-specific performance metrics of the RF classifier are summarized in Table 5. The model showed consistent and robust classification across all phylogenetic categories. The iHGT and NoHGT classes achieved the highest accuracy; notably, the minority NoHGT class (n = 73) performed comparably to the larger classes, confirming that performance was not driven by class size. The Inconclusive subclasses—Patchy phylogeny and Multiple MC—showed lower F1-scores, reflecting their greater structural and biological similarity. Overall, macro-F1 and balanced accuracy both reached 0.893, with narrow confidence intervals across all classes confirming stable performance over repeated validation runs.

**TABLE 5:** Class-specific classification performance of the Random Forest model across the five phylogenetic pattern categories.

Class	Support	Precision	Recall	F1-score
iHGT	332	0.9855 [0.9805, 0.9906]	0.9825 [0.9780, 0.9870]	0.9839 [0.9804, 0.9873]
NoHGT	73	0.9761 [0.9646, 0.9877]	0.9741 [0.9637, 0.9843]	0.9742 [0.9669, 0.9815]
Limited donor evidence	117	0.9391 [0.9271, 0.9511]	0.9487 [0.9336, 0.9638]	0.9427 [0.9327, 0.9528]
Patchy phylogeny	314	0.8352 [0.8253, 0.8450]	0.8653 [0.8518, 0.8787]	0.8488 [0.8416, 0.8560]
Multiple MC	176	0.7511 [0.7336, 0.7686]	0.6927 [0.6704, 0.7151]	0.7169 [0.7034, 0.7305]

This table summarizes class-specific performance metrics of the RF, classifier using repeated stratified cross-validation. Precision, recall, and F1-score are reported for each phylogenetic pattern category as the mean across 50 validation folds (5-fold cross-validation repeated 10 times), together with their corresponding 95% confidence intervals. Support indicates the total number of instances belonging to each class in the complete training dataset.

Figure 5 provides a comprehensive visual assessment of classifier performance and robustness. The AUC-ROC curves (panel A) and confusion matrix (panel B) confirm excellent discriminative ability across all five classes, with the lowest performance observed for Patchy phylogeny and Multiple MC, consistent with the higher misclassification rates between these structurally similar categories. A follow-up analysis of *p_recipientMC* values showed that iHGT/Limited donor evidence confusions were concentrated in the 0.74–0.86 range, representing a natural decision boundary where trees from both classes become structurally indistinguishable. Pairwise comparisons using the Nemenyi *post hoc* test confirmed that neither imbalance-handling strategy outperformed the baseline RF: the class-weighted variant performed significantly worse in macro-F1 (p = 0.0076), while SMOTE produced comparable performance (p = 0.3675), indicating that the baseline model is robust to class imbalance without requiring resampling or adjusted class weights (panel C). As all evaluated metrics showed consistent trends, only macro-F1 is reported; full summary statistics and raw fold-level values are provided in Supplementary Table S14. The learning curve (panel D) shows that cross-validation balanced accuracy increased progressively with training set size and stabilized above approximately 480 samples, confirming that the available dataset was sufficient for stable generalization.

**FIGURE 5:**
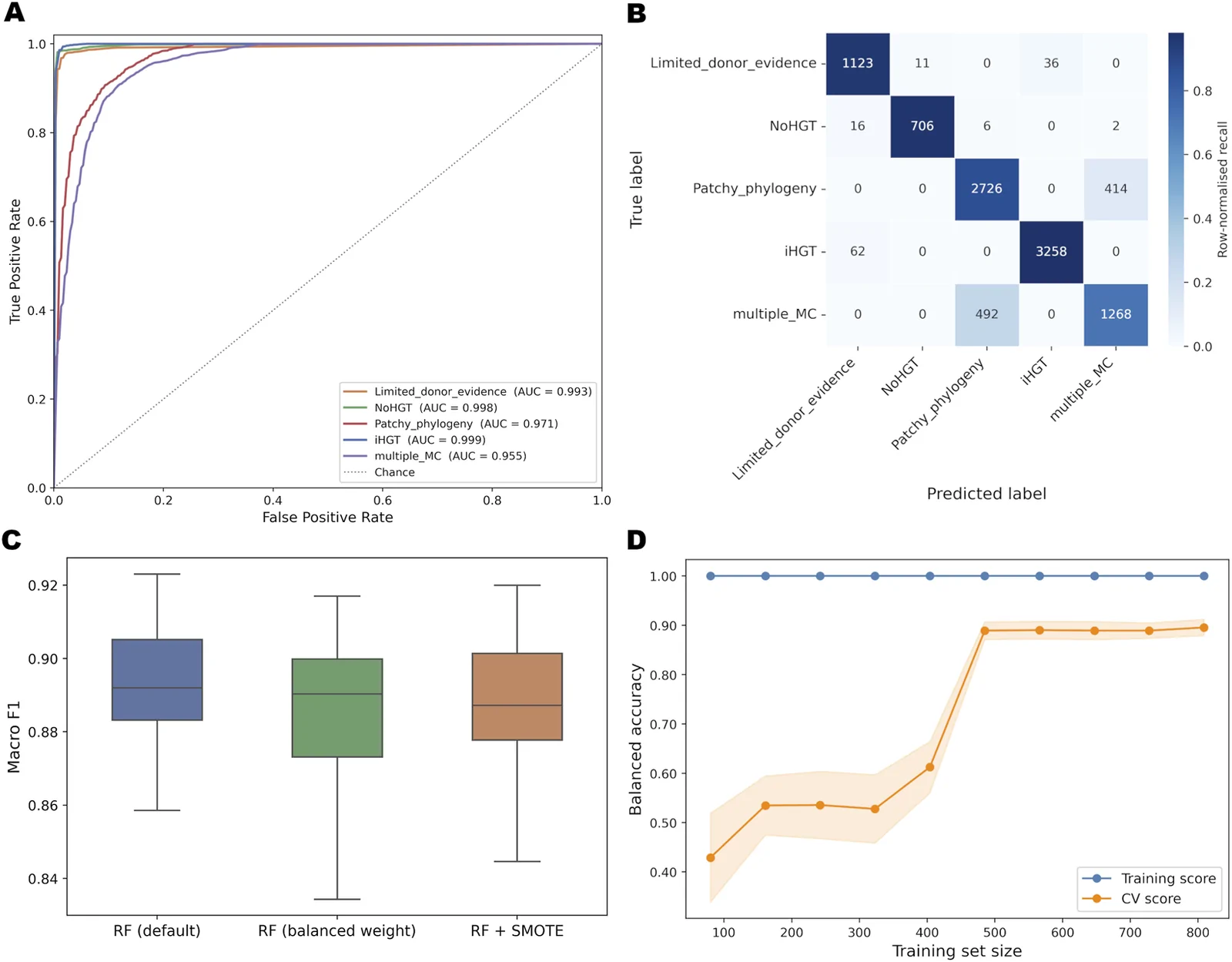
Comprehensive evaluation of Random Forest classifier performance and robustness. **(A)** Multiclass ROC curves using a one-vs-rest approach; AUC values are shown in the legend. **(B)** Summed confusion matrix across all cross-validation repetitions (5-fold × 10 repeats); rows represent true labels, columns predicted labels. **(C)** Comparison of macro-F1 across three model variants: baseline RF, class-weighted RF, and RF trained on SMOTE-resampled data. Boxplots show the distribution across 50 validation folds; letters indicate groups with no statistically significant difference (Friedman test, Nemenyi *post hoc*). **(D)** Learning curve showing training and cross-validation balanced accuracy as a function of training set size; shaded regions represent standard deviation across folds.

### 3.3 Feature importance and model interpretability

To evaluate whether the Random Forest classifier captured predictive information beyond the explicit criteria used to define the phylogenetic patterns, a combined rule-based comparison and feature ablation analysis was performed (Figure 6A). The rule-based baseline (Decision tree), the full RF model, and four feature-reduced RF variants were evaluated under the same repeated stratified cross-validation protocol; a Friedman test with Nemenyi *post hoc* correction identified four statistically distinct performance groups (p < 0.05; pairwise p-values in Supplementary Table S16). As before, only macro-F1 is reported; full summary statistics and raw fold-level values are provided in Supplementary Table S15. The full RF and the without-n_aMCs variant formed the top-performing group (b), significantly outperforming all others including the rule-based baseline (a), which in turn exceeded the clade-composition-only configuration (d). Configurations excluding proportion-based features or relying exclusively on distance descriptors formed the lowest-performing group (c), confirming that taxonomic composition features are the primary drivers of classification accuracy. The superior performance of the full RF over the rule-based baseline indicates that the classifier captures non-linear feature relationships beyond what can be recovered through deterministic rules, particularly for ambiguous and underrepresented phylogenetic patterns.

**FIGURE 6:**
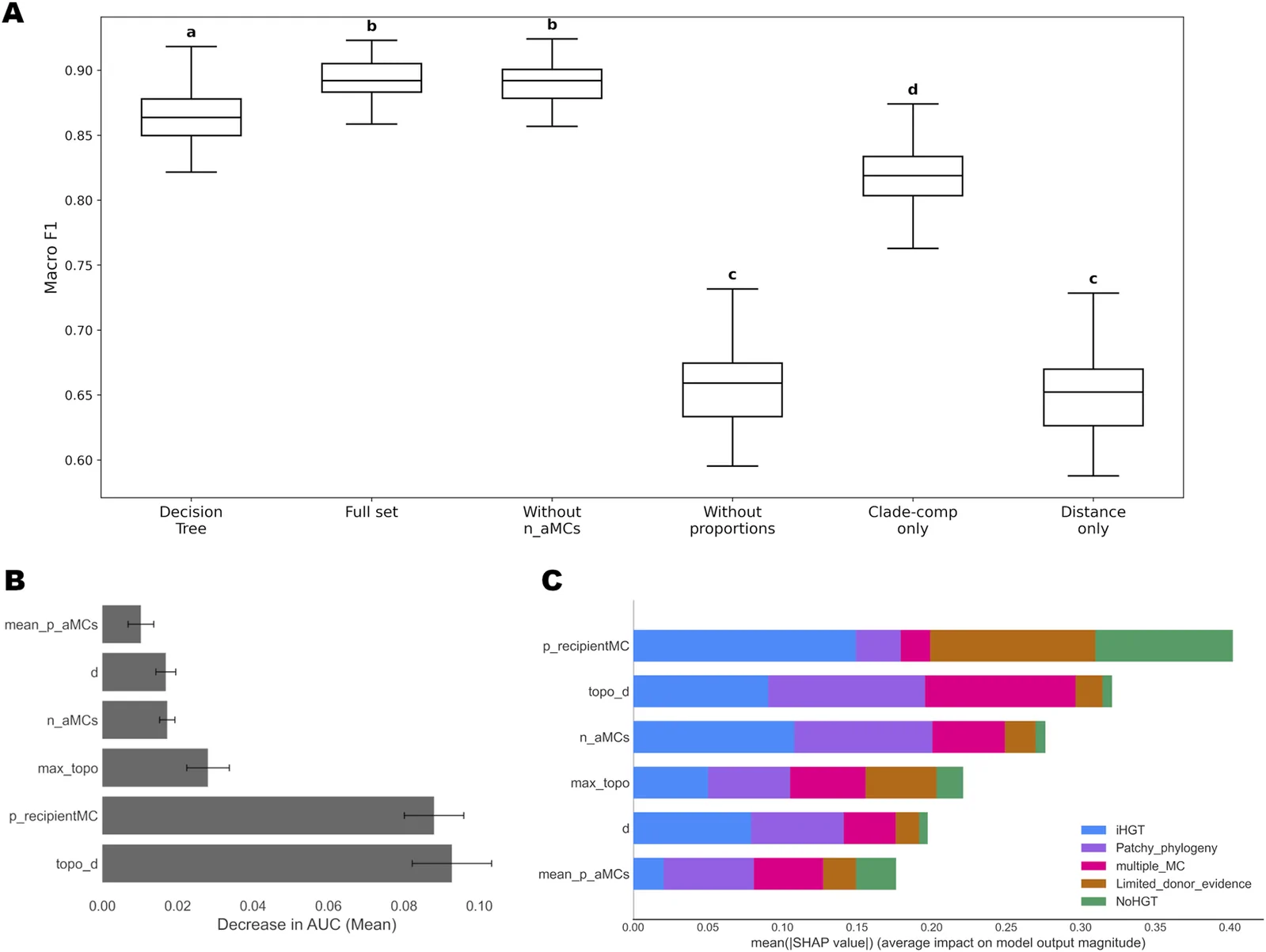
Feature importance analysis for the Random Forest classifier. **(A)** Distribution of macro-F1 scores across 50 validation folds for each feature configuration evaluated in the ablation analysis; the rule-based baseline is included as a reference. Letters above boxes indicate statistically distinct groups (Nemenyi *post hoc*, α = 0.05). **(B)** Permutation feature importance estimated as the mean decrease in weighted multiclass AUC after randomly permuting each feature in the test set; error bars indicate standard deviation across permutations. **(C)** SHAP-based feature importance showing mean absolute SHAP values per feature; bar colors represent the relative contribution of each phylogenetic class to the total SHAP value.

Permutation importance and SHAP analyses (Figures 6B,C) converged on a consistent picture of feature contributions at the individual level. Both analyses identified *topo_d* and *p_recipientMC* as the most influential predictors: permuting either feature produced the largest decreases in weighted multiclass AUC, and both features showed the highest overall SHAP values. Class-specific SHAP contributions revealed that *p_recipientMC* primarily influenced discrimination of Limited donor evidence, NoHGT, and iHGT patterns, while *topo_d* and *d* were most relevant for separating Multiple MC from Patchy phylogeny, the most challenging classification boundary. *n_aMCs* contributed substantially to separating iHGT from the Inconclusive classes, and *max_topo* and *mean_p_aMCs* showed moderate but consistent contributions across multiple categories. Together, the ablation, permutation, and SHAP analyses indicate that the classifier relies on complementary contributions from taxonomic composition and topological descriptors, with both feature groups necessary to achieve accurate classification across the full spectrum of phylogenetic patterns.

### 3.4 Benchmarking of the Random Forest classifier and external validation

The RF model was applied to phylogenetic trees from two independent validation datasets: a simulated dataset under controlled conditions and an unseen real biological dataset. The model achieved low misclassification rates in both settings (7.8% and 10.43%, respectively; Supplementary Tables S10–S11), with the modest increase for empirical data reflecting the greater complexity and variability of real biological sequences. In both cases, the dominant source of error was confusion between Multiple MC and Patchy phylogeny patterns, consistent with the classification boundary identified during cross-validation.

When benchmarked against AVP, Alien Index, and HGT Index, the RF consistently outperformed all comparison methods on every evaluated metric across both datasets (Figure 7, panels A–B; detailed results in Supplementary Tables S12-S13). Among the alternative tools, AVP showed the strongest overall performance, with better recall and MCC than HGT Index. The Alien Index performed poorest in both settings, yielding negative MCC values indicating failure to reliably detect iHGT events.

**FIGURE 7:**
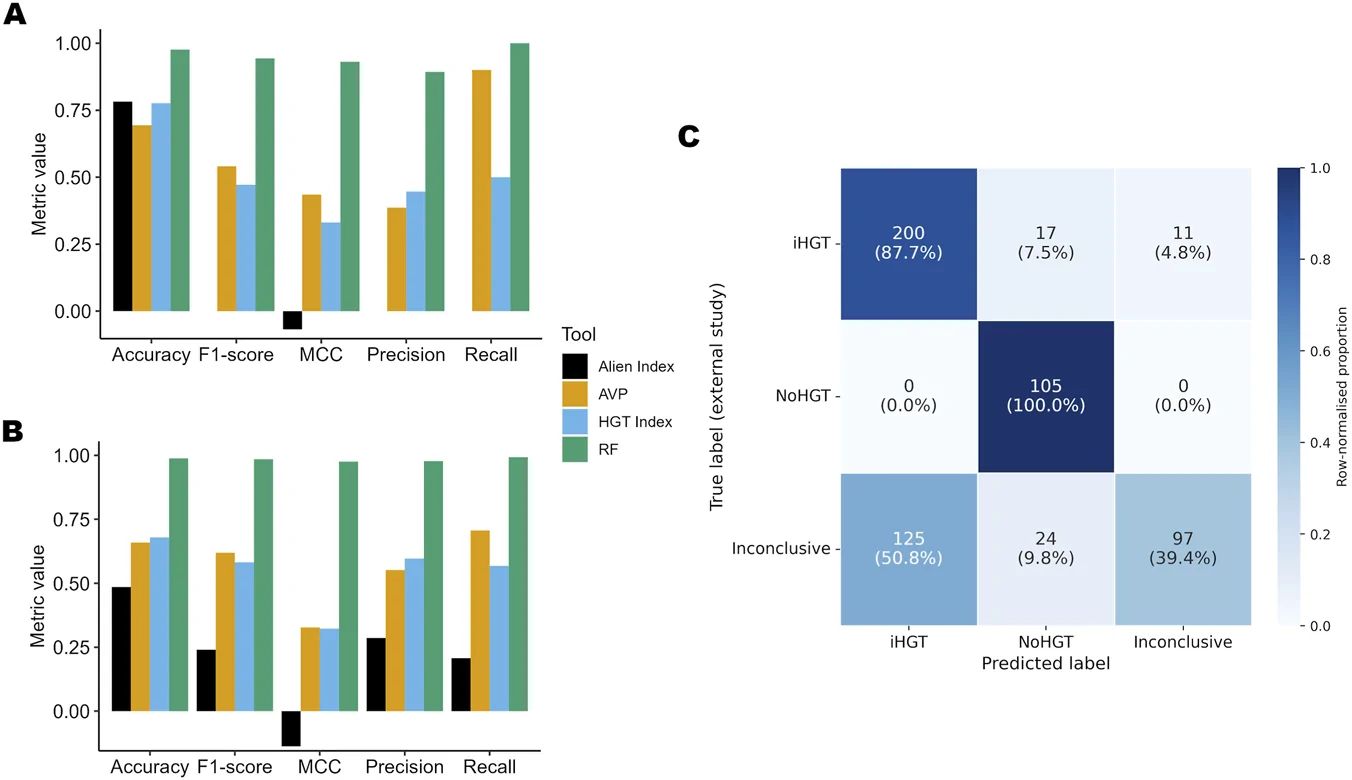
Benchmarking and external validation of the Random Forest (RF) classifier. **(A,B)** Comparison of classification performance between the RF model, AVP, Alien Index, and HGT Index on the simulated dataset **(A)** and the real biological dataset **(B)**; performance was evaluated using accuracy, F1-score, Matthews correlation coefficient (MCC), precision, and recall. **(C)** Agreement between the RF classifier and independent annotations from Crisp et al. (2015) and Ciach et al. (2024) across 579 phylogenetic candidates; rows represent reference categories assigned by the external studies and columns represent RF-predicted categories, with row-normalized proportions shown in each cell.

The RF model was further evaluated against independently annotated data from Crisp et al. (2015) and Ciach et al. (2024), covering 579 phylogenetic trees annotated by independent research groups using different methodological frameworks (Figure 7C; feature values and classifications in Supplementary Table S17). Classification of iHGT and NoHGT candidates showed strong agreement with external annotations, with perfect recall for NoHGT. Discordance was concentrated in the Inconclusive category, where the RF model frequently assigned an iHGT classification, reflecting differences in how borderline cases are handled across distinct methodological criteria. Overall agreement across the independent dataset was 69.4%.

## 4 Discussion

While several automated approaches exist for iHGT candidate detection, the classification of phylogenetic tree topologies into biologically meaningful evolutionary patterns has traditionally depended on expert manual inspection, a process that is inherently subjective, difficult to reproduce, and challenging to scale to large datasets. As genomic data continues to expand, there is a growing need for approaches that can formalize expert-driven interpretation into reproducible and quantitative frameworks. In this study, we address this challenge by introducing a computational framework that translates recurrent phylogenetic patterns into a supervised classification problem. By combining biologically informed feature extraction with machine-learning approaches, we provide a scalable and interpretable method for classifying evolutionary scenarios associated with iHGT.

A central contribution of this work is the formalization of phylogenetic tree interpretation into a set of discrete, biologically meaningful patterns. Rather than restricting the analysis to a binary distinction between HGT and non-HGT, the proposed framework distinguishes among five recurrent evolutionary scenarios, including cases where the phylogenetic signal is insufficient to support definitive conclusions. This multi-class perspective more accurately reflects the complexity of evolutionary processes, where alternative explanations such as differential gene loss, incomplete lineage sorting, or multiple transfer events may generate similar topological signals (Bremer et al., 2025; Armijos-Jaramillo and Aguirre-Carvajal, 2026). By explicitly incorporating these ambiguous cases into an Inconclusive category (Limited donor evidence, Multiple MC and Patchy phylogeny), the framework avoids overinterpretation and provides a more nuanced representation of phylogenetic evidence.

The ablation, permutation, and SHAP analyses collectively indicate that the RF classifier benefits from both taxonomic composition and distance-based descriptors of phylogenetic structure. Among the evaluated features, *topo_d* and *p_recipientMC* emerged as the most influential predictors. The feature *topo_d* captures the structural proximity of the query to lineages outside the inferred donor-recipient relationship: small topological distances indicate that the query clusters closely with additional major clades, consistent with the Multiple MC scenario in which homologs from several lineages form a monophyletic group, a configuration often better explained by vertical inheritance followed by differential gene loss (Bremer et al., 2025). Larger topological distances, by contrast, indicate that additional major clades are distributed across different regions of the tree, characteristic of Patchy phylogenies where discontinuous distributions may reflect multiple independent HGT events, gene loss, or incomplete lineage sorting (Galtier and Daubin, 2008; Stolzer et al., 2012; Davidson et al., 2015; Steenwyk et al., 2023).

Clade-composition features, particularly *p_recipientMC*, complement *topo_d* by capturing the relative representation of recipient taxa in the tree. The balance between donor and recipient sequences is a widely used indicator of HGT directionality (Podell and Gaasterland, 2007; Ravenhall et al., 2015; Kloub et al., 2021). iHGT scenarios are typically characterized by a well-represented donor clade relative to the recipient, whereas Limited donor evidence patterns involve a sparse representation of donor sequences, which reduces confidence in inferring the direction of transfer (Schmitt and Lumbsch, 2009; Aguirre-Carvajal and Armijos-Jaramillo, 2026).

The number of additional major clades (*n_aMCs*) further informs whether complex evolutionary scenarios warrant consideration. When *n_aMCs* = 0, the phylogeny is restricted to a two-clade structure and the model focuses on distinguishing iHGT from NoHGT using proportional features, while the presence of one or more additional clades guides classification toward Inconclusive patterns. Together, these features capture complementary aspects of phylogenetic structure enabling the model to distinguish among a broad spectrum of evolutionary scenarios while maintaining biological interpretability. These features capture complementary aspects of phylogenetic structure: local topology, global lineage diversity, and clade representation.

Tree-based ensemble methods, RF and XGBoost, consistently achieved the highest predictive performance, likely reflecting their ability to model complex, non-linear interactions among phylogenetically derived features that are poorly suited to methods assuming linearity or feature independence, such as Logistic Regression and Gaussian Naïve Bayes (Breiman, 2001; Hastie et al., 2009; Pensa et al., 2025). Neural network approaches may additionally be constrained by the modest dataset size. RF was selected over XGBoost for its greater interpretability: the independent construction of trees in RF facilitates transparent and stable feature importance estimation, whereas the sequential nature of XGBoost introduces more complex dependencies that are less straightforward to interpret at a global level (Rynkiewicz, 2012; Chen and Guestrin, 2016; Lundberg and Lee, 2017). Notably, hyperparameter optimization did not lead to significant improvements, indicating that the extracted features encode a strong and consistent phylogenetic signal that can be reliably learned without extensive parameter tuning (Oshiro et al., 2012; Probst et al., 2019).

An important consideration is the relatively modest training dataset size and unequal class representation, particularly for the NoHGT class. However, several lines of evidence suggest these factors did not significantly affect model performance: class-specific precision, recall, and F1-scores were high across all categories including NoHGT; macro-F1 and balanced accuracy closely matched their weighted equivalents; and class-weighting and SMOTE-based resampling produced only marginal changes. These findings are consistent with the moderate imbalance ratio of the dataset (4.5:1), as machine-learning performance is generally more sensitive to severe imbalance exceeding 10:1 or 100:1 (He and Garcia, 2009; Haixiang et al., 2017; Leevy et al., 2018).

The learning curve analysis provides additional evidence regarding the adequacy of the available training data. Although the Random Forest classifier achieved near-perfect performance on the training subsets, this behavior is commonly observed in Random Forest models due to their use of deep, largely unpruned decision trees, and should not be interpreted as evidence of poor generalization by itself (Barreñada et al., 2024). More informative is the behavior of the cross-validation performance, which increased progressively with training set size and approached a stable plateau at approximately 0.89 balanced accuracy. Moreover, the variability of the cross-validation estimates decreased as additional training examples were incorporated. Together, these patterns suggest that the model learned reproducible relationships from the data rather than relying on a small subset of observations and that the current dataset is sufficient to achieve stable performance (Géron, 2022). This interpretation is further supported by the external validation analysis based on 579 independently annotated candidates from Crisp et al. (2015) and Ciach et al. (2024), in which the RF classifier showed substantial agreement with original annotations despite being applied to data generated independently of the training framework.

Additional evidence of the model’s utility was provided by the benchmarking analysis, in which the RF classifier was compared against AVP, Alien Index, and HGT Index. The RF model consistently outperformed all three methods. The performance gap between the RF classifier and the similarity-based methods (Alien Index and HGT Index) reflects a fundamental methodological difference: whereas the RF framework explicitly models phylogenetic tree structure, these approaches quantify sequence similarity between donor and recipient taxa and therefore cannot capture the topological signal that defines the phylogenetic patterns used in this study. This limitation was most evident in the Alien Index, which failed to detect the majority of true iHGT candidates while simultaneously generating a substantial number of false positives, producing a negative MCC in the benchmark analysis. Although both Alien Index and HGT Index compare the best BLAST hit against non-recipient vs. recipient taxa, they differ in the similarity measure used: the Alien Index is based on E-value ratios on a logarithmic scale (Gladyshev et al., 2008), while the HGT Index uses raw bitscore differences (Boschetti et al., 2012). This difference in scoring scale likely contributed to the higher sensitivity of the HGT Index in detecting iHGT candidates. Several studies have highlighted the limitations of such similarity-based approaches. For instance, processes such as amelioration can obscure ancient horizontal gene transfer events by homogenizing sequence composition over time, reducing detectable differences between donor and recipient lineages (Boto, 2009). Conversely, high sequence similarity does not necessarily reflect true evolutionary relatedness, as it may arise from convergent evolution or uneven taxonomic sampling (Koski and Golding, 2001; Joshi and Xu, 2007; Aguirre-Carvajal et al., 2024).

AVP, a phylogenetically explicit method, outperformed both similarity-based approaches but remained substantially below the RF classifier. This performance gap likely reflects a fundamental difference in the scope of phylogenetic information considered: AVP restricts its analysis to two branches adjacent to the query sequence, the sister branch and the ancestral sister branch (Koutsovoulos et al., 2022), whereas the proposed framework extracts features from the entire tree topology, capturing topological distances, lineage diversity, and clade distributions across all terminal nodes. Although this local focus reduces computational load, it may obscure evolutionary signals encoded in remote branches, including patterns of gene loss, incomplete lineage sorting, or multi-clade distributions that would otherwise support alternative evolutionary hypotheses or challenge iHGT inferences. The global perspective of the RF classifier enables systematic characterization of the full range of phylogenetic patterns, reducing the risk of misclassifying scenarios that share local topological signatures but differ in their broader tree structure.

The detection of horizontal gene acquisition in eukaryotes has generated a variety of tools adopting parametric, sequence similarity-based, and explicit phylogenetic approaches (Boto, 2009; Ravenhall et al., 2015; Dutton and Reiter, 2023). Although gene tree-species tree reconciliation is considered one of the most rigorous phylogenetic approaches for iHGT detection (Richards et al., 2011), reconciliation artifacts, database bias, taxon sampling and methodological decisions can all contribute to false inferences (Dupont and Cox, 2017; Aguirre-Carvajal and Armijos-Jaramillo, 2026), highlighting the absence of consensus on confident iHGT identification criteria. This was directly evidenced in the external validation analysis: discordance between the RF classifier and Ciach et al. (2024) was concentrated in the Inconclusive category, where our model assigned iHGT to trees that Ciach et al. labeled Inconclusive due to weak bootstrap support. This illustrates how different methodological criteria applied to the same trees yield different classifications, reinforcing the need for transparent and explicitly defined inference criteria. Supervised machine learning frameworks represent a promising means of addressing this variability by formalizing classification criteria into explicit, reproducible, data-driven decision boundaries; however, to our knowledge, no method has previously applied this approach within a fully phylogenetic framework for iHGT inference (Wijaya et al., 2025).

The RF model exhibits its greatest classification difficulty at the boundary between Multiple MC and Patchy phylogeny. Although both patterns belong to the Inconclusive group, their differentiation depends on whether additional major clades form a cohesive cluster or are dispersed across different regions of the phylogeny. Capturing such higher-order topological relationships using scalar descriptors is inherently challenging, as it requires sensitivity to global tree structure rather than simple taxonomic composition or pairwise distance measurements (Zhang, 2023; Xie et al., 2025). Future developments may benefit from graph representation learning and transformer-based architectures, which can capture topological relationships and hierarchical dependencies more comprehensively than summary descriptors, potentially improving discrimination between these closely related scenarios (Zhang, 2023; Iranzad and Liu, 2025; Mu et al., 2025).

A further consideration concerns the potential conceptual overlap between class labels and training features, as both derive from the same underlying phylogenetic information. However, this should not be interpreted as information leakage, since labels were assigned through expert visual inspection and not generated from the quantitative features themselves; similar approaches are common in supervised machine-learning applications in biology (Valizadegan et al., 2013; Libbrecht and Noble, 2015). The Decision Tree baseline, which recovers a substantial proportion of the classification signal using optimized thresholds, confirms that part of the predictive performance reflects expert annotation logic. However, the superior performance of the RF and the ablation results indicate that classification cannot be explained solely by these explicit rules.

Finally, an important contribution of this study is the development of a validation resource for benchmarking iHGT detection methods. To date, there is no widely accepted reference dataset for robust evaluation in this domain (Wijaya et al., 2025). The simulations indicate that certain topological configurations commonly interpreted as evidence of iHGT may require a series of highly specific and biologically unlikely events, as highlighted by Bremer et al. (2025). For instance, reproducing the Multiple MC pattern required simulating independent gene transfers from bacteria into multiple eukaryotic major clades simultaneously, and generating Patchy phylogenies required multiple independent transfers involving different bacterial donors and distinct eukaryotic recipients. These simulations suggest that interpreting such complex topologies as direct evidence of iHGT may overestimate the likelihood of these events.

This study demonstrates that phylogenetic tree interpretation can be formalized into a consistent, data-driven process. Despite these advantages, the tool has inherent limitations tied to current knowledge and evaluation criteria. Future developments should focus on expanding training data diversity, incorporating additional evolutionary evidence, and further resolving ambiguous phylogenetic patterns to establish more robust standards for iHGT detection in large-scale comparative genomics.

## 5 Conclusion

In this study, we present a reproducible and interpretable framework for the automated classification of expert-defined phylogenetic patterns relevant to the evaluation of interkingdom horizontal gene transfer (iHGT) candidates. By formalizing expert-driven phylogenetic interpretation into a supervised machine-learning problem, we demonstrate that complex evolutionary scenarios can be systematically represented using a small set of biologically meaningful features derived from gene tree topology and taxonomic composition.

Our results demonstrate that topological descriptors, particularly those reflecting the relative placement of additional major clades, are the most informative for distinguishing among evolutionary patterns. When combined with measures of lineage diversity and clade representation, these features enable accurate classification across five expert-defined categories, including ambiguous cases often overlooked in binary iHGT frameworks.

The framework achieved high classification performance and strong agreement with reference annotations across simulated and real biological datasets, maintained substantial agreement on an independently annotated dataset, and achieved better results relative to existing approaches, highlighting the advantages of incorporating global tree structure into the automated classification of phylogenetic patterns. At the same time, the analysis underscores that certain phylogenetic patterns commonly interpreted as evidence of iHGT may arise from alternative evolutionary processes, emphasizing the need for cautious and context-aware interpretation.

This work demonstrates a general strategy for translating qualitative phylogenetic reasoning into quantitative, scalable, and reproducible models. As genomic datasets continue to expand, frameworks that combine phylogenetic rigor with machine-learning scalability will be essential for enabling robust evolutionary inference and for developing standardized criteria in comparative genomics.

## Data Availability

The datasets presented in this study can be found in online repositories. The names of the repository/repositories and accession number(s) can be found in the article/Supplementary Material.
