# Supplementary material for: A machine learning framework for interpreting phylogenetic tree patterns in interkingdom horizontal gene transfer
Source: Front Bioinform. 2026 Sep 21;6:1891949. doi: 10.3389/fbinf.2026.1891949 (PMC13634943; doi:10.3389/fbinf.2026.1891949)
Supplement: Supplementary file 1 [file DataSheet1.pdf]

Section 1. Detailed Feature Extraction Pipeline

All features included in the model were derived directly from phylogenetic trees using a fully reproducible pipeline implemented in Python v3.10.12 (Figure S1). The pipeline accepts trees in either NEXUS or Newick format, and branch lengths are expressed in substitutions per site. Phylogenies were parsed using the *Phylo* module of Biopython v1.85 (Cock *et al.*, 2009).

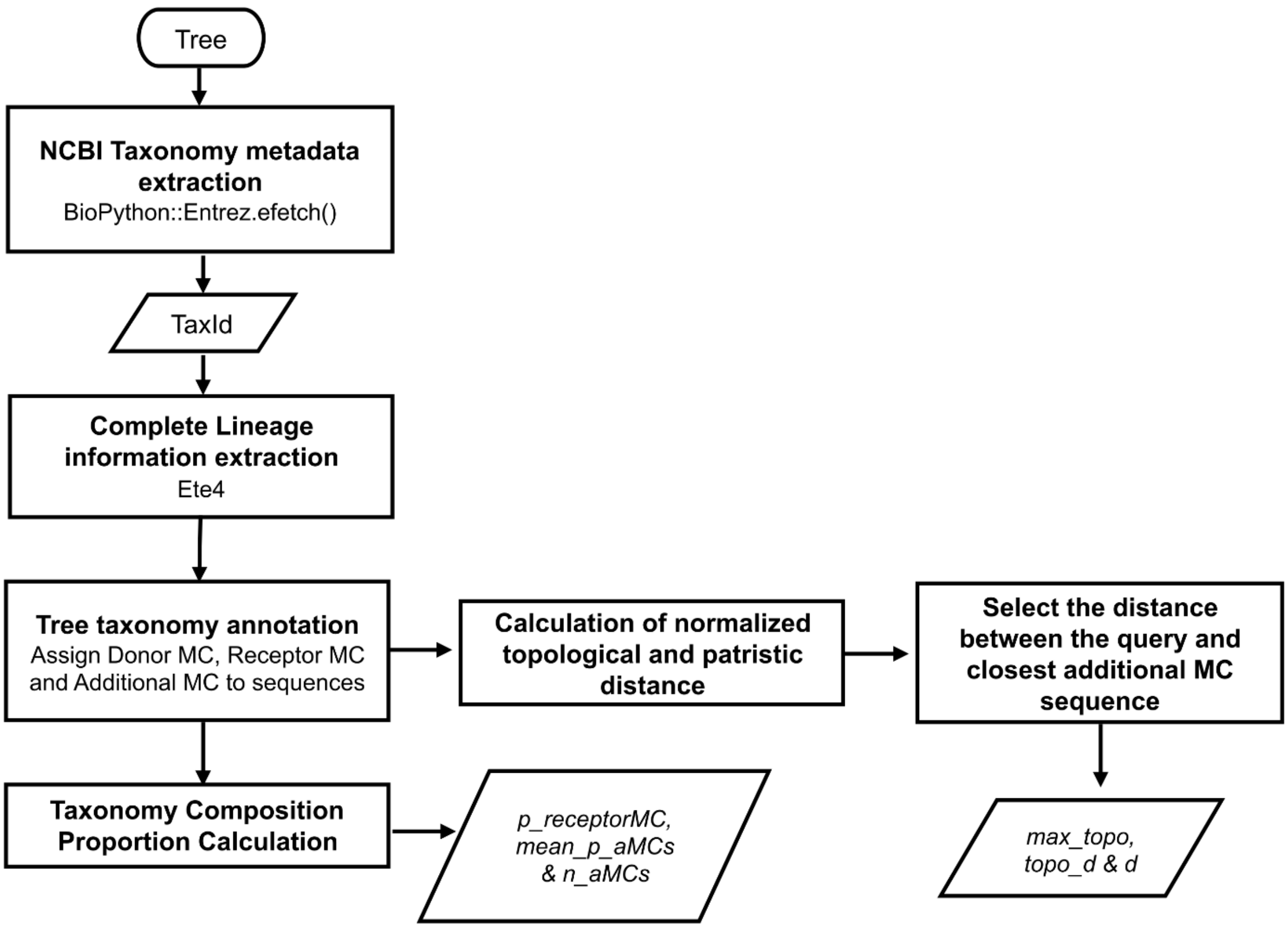

Figure S1. Overview of the workflow used for taxonomic annotation and extraction of phylogenetic features. The workflow begins with a phylogenetic tree, from which NCBI Taxonomy metadata are retrieved from NCBI sequence identifiers to obtain taxonomic identifiers. Complete lineage information is then extracted using ETE4, enabling detailed taxonomic annotation of each sequence. Based on this information, each sequence is assigned to the donor, recipient, or an additional major clade (MC). Next, normalized patristic and topological distances are computed between the query sequence and all other sequences in the tree. From these values, the minimum distance between the query and sequences from additional MCs is retained, yielding the features *d* and *topo_d*. The maximum topological distance within the tree (*max_topo*) is also calculated. Finally, taxonomic composition features are computed, including the proportions of sequences belonging to recipient MC (*p_recipientMC*), as well as the mean proportion and number of additional MCs (*mean_p_aMCs* and *n_aMCs*). Together, these variables constitute the feature set used for downstream classification of phylogenetic patterns.

Each terminal node corresponds to an NCBI protein accession, enabling retrieval of associated taxonomic metadata. For each accession, the corresponding GenBank record was obtained using the Entrez efetch utility implemented in Biopython. The NCBI taxonomy identifier (TaxID) was extracted from the record and used to retrieve the complete taxonomic lineage through the NCBITaxa module of the ETE Toolkit v4.0.0b2 (Huerta-Cepas, Serra and Bork, 2016).

Based on the retrieved taxonomic lineages, sequences were assigned to their corresponding major and minor clades according to the classification scheme summarized in Table 2. Following this classification, all terminal taxa in each tree were grouped into three analytical categories: (i) sequences belonging to the major recipient clade, (ii) sequences assigned to the putative major donor clade, and (iii) sequences affiliated with additional major clades.

To define the donor major clade (MC), the recipient MC—provided by the user—is first excluded from the set of MCs present in the tree, after which the most abundant remaining MC is selected as the donor MC. Following taxonomic annotation, patristic and topological distances were computed using the Phylo module of Biopython and normalized by the corresponding tree diameter. For each distance metric, the minimum distance between the query sequence and sequences from additional MCs was retained, yielding the features *d* and *topo_d*. Additionally, the maximum topological distance within the tree (*max_topo*) was extracted.

Finally, features describing the taxonomic composition of each tree were computed. Specifically, the proportions of recipient (*p_recipientMC*) major clades were estimated. For additional MCs, individual proportions were computed and used to derive *mean_p_uMCs* and *n_uMCs*. All proportion calculations were restricted to sequences for which a complete taxonomic lineage could be successfully retrieved and assigned.

The resulting feature set was compiled into a tab-delimited output file representing the final output of the pipeline. The parameters used to run the pipeline are summarized in Supplementary Table 1.

Section 2. Decision Tree Hyperparameter Selection

The optimal tree depth for the rule-based Decision Tree baseline was selected through nested cross-validation. An inner RepeatedStratifiedKFold loop (n_splits=5, n_repeats=3, random_state=0) was used to evaluate candidate maximum depths of 3, 4, 5, 6, 7, and unconstrained growth, using macro-F1 as the optimization criterion. The optimal depth was 7, yielding an inner cross-validation macro-F1 of 0.8626. The Decision Tree with this depth was then evaluated under the same outer cross-validation protocol as the Random Forest (RepeatedStratifiedKFold, n_splits=5, n_repeats=10, random_state=42), ensuring that comparisons between both classifiers were based on identical data partitions.
