# Supplementary material for: A machine learning framework for interpreting phylogenetic tree patterns in interkingdom horizontal gene transfer
Source: Front Bioinform. 2026 Sep 21;6:1891949. doi: 10.3389/fbinf.2026.1891949 (PMC13634943; doi:10.3389/fbinf.2026.1891949)
Supplement: Supplementary file 2 [file DataSheet2.pdf]

Decision Tree Classification Rules Learned on the Full Training Dataset

Decision Tree (max_depth=7) trained on full dataset

Classes: ['Limited_donor_evidence', 'NoHGT', 'Patchy_phylogeny', 'iHGT', 'multiple_MC']

```
|--- d <= -0.50
|   |--- p_recipientMC <= 0.75
|   |   |--- max_topo <= 22.00
|   |   |   |--- class: 0
|   |   |--- max_topo >  22.00
|   |   |   |--- max_topo <= 64.50
|   |   |   |   |--- p_recipientMC <= 0.56
|   |   |   |   |   |--- class: 3
|   |   |   |   |--- p_recipientMC >  0.56
|   |   |   |   |   |--- class: 0
|   |   |   |--- max_topo >  64.50
|   |   |   |   |--- max_topo <= 133.00
|   |   |   |   |   |--- max_topo <= 129.00
|   |   |   |   |   |   |--- class: 3
|   |   |   |   |   |--- max_topo >  129.00
|   |   |   |   |   |   |--- p_recipientMC <= 0.27
|   |   |   |   |   |   |   |--- class: 3
|   |   |   |   |   |   |--- p_recipientMC >  0.27
|   |   |   |   |   |   |   |--- class: 0
|   |   |   |   |--- max_topo >  133.00
|   |   |   |   |   |--- class: 3
|   |--- p_recipientMC >  0.75
|   |   |--- p_recipientMC <= 1.00
|   |   |   |--- max_topo <= 511.50
|   |   |   |   |--- p_recipientMC <= 0.77
```

```
|   |   |   |   |   |--- max_topo <= 73.50
|   |   |   |   |   |   |--- class: 0
|   |   |   |   |   |--- max_topo >  73.50
|   |   |   |   |   |   |--- class: 3
|   |   |   |   |--- p_recipientMC >  0.77
|   |   |   |   |   |--- max_topo <= 200.00
|   |   |   |   |   |   |--- p_recipientMC <= 0.81
|   |   |   |   |   |   |   |--- class: 0
|   |   |   |   |   |   |--- p_recipientMC >  0.81
|   |   |   |   |   |   |   |--- class: 0
|   |   |   |   |   |--- max_topo >  200.00
|   |   |   |   |   |   |--- max_topo <= 204.50
|   |   |   |   |   |   |   |--- class: 1
|   |   |   |   |   |   |--- max_topo >  204.50
|   |   |   |   |   |   |   |--- class: 0
|   |   |   |--- max_topo >  511.50
|   |   |   |   |--- p_recipientMC <= 0.97
|   |   |   |   |   |--- max_topo <= 523.50
|   |   |   |   |   |   |--- max_topo <= 522.00
|   |   |   |   |   |   |   |--- class: 3
|   |   |   |   |   |   |--- max_topo >  522.00
|   |   |   |   |   |   |   |--- class: 0
|   |   |   |   |   |--- max_topo >  523.50
|   |   |   |   |   |   |--- class: 3
|   |   |   |   |--- p_recipientMC >  0.97
|   |   |   |   |   |--- class: 0
|   |   |--- p_recipientMC >  1.00
|   |   |   |--- class: 1
|--- d >  -0.50
```

```
|   |--- topo_d <= 0.14
|   |   |--- mean_p_aMCs <= 0.00
|   |   |   |--- max_topo <= 378.00
|   |   |   |   |--- class: 1
|   |   |   |--- max_topo >  378.00
|   |   |   |   |--- topo_d <= 0.06
|   |   |   |   |   |--- class: 4
|   |   |   |   |--- topo_d >  0.06
|   |   |   |   |   |--- d <= 0.64
|   |   |   |   |   |   |--- p_recipientMC <= 0.01
|   |   |   |   |   |   |   |--- class: 2
|   |   |   |   |   |   |--- p_recipientMC >  0.01
|   |   |   |   |   |   |   |--- class: 2
|   |   |   |   |   |--- d >  0.64
|   |   |   |   |   |   |--- class: 4
|   |   |--- mean_p_aMCs >  0.00
|   |   |   |--- max_topo <= 521.00
|   |   |   |   |--- p_recipientMC <= 0.00
|   |   |   |   |   |--- topo_d <= 0.10
|   |   |   |   |   |   |--- class: 4
|   |   |   |   |   |--- topo_d >  0.10
|   |   |   |   |   |   |--- class: 2
|   |   |   |   |--- p_recipientMC >  0.00
|   |   |   |   |   |--- d <= 0.12
|   |   |   |   |   |   |--- p_recipientMC <= 0.48
|   |   |   |   |   |   |   |--- class: 4
|   |   |   |   |   |   |--- p_recipientMC >  0.48
|   |   |   |   |   |   |   |--- class: 2
|   |   |   |   |   |--- d >  0.12
```

```
| | | | | | |--- class: 4
| | | |--- max_topo >  521.00
| | | | |--- topo_d <= 0.09
| | | | | |--- n_aMCs <= 1.50
| | | | | | |--- d <= 0.09
| | | | | | | |--- class: 4
| | | | | | |--- d >  0.09
| | | | | | | |--- class: 2
| | | | | |--- n_aMCs >  1.50
| | | | | | |--- p_recipientMC <= 0.16
| | | | | | | |--- class: 4
| | | | | | |--- p_recipientMC >  0.16
| | | | | | | |--- class: 2
| | | | |--- topo_d >  0.09
| | | | | |--- max_topo <= 550.50
| | | | | | |--- d <= 0.42
| | | | | | | |--- class: 4
| | | | | | |--- d >  0.42
| | | | | | | |--- class: 2
| | | | | |--- max_topo >  550.50
| | | | | | |--- p_recipientMC <= 0.00
| | | | | | | |--- class: 4
| | | | | | |--- p_recipientMC >  0.00
| | | | | | | |--- class: 2
| |--- topo_d >  0.14
| | |--- max_topo <= 423.00
| | | |--- topo_d <= 0.48
| | | | |--- max_topo <= 69.50
| | | | | |--- d <= 0.71
```

```
| | | | | | |--- max_topo <= 28.00
| | | | | | | |--- class: 4
| | | | | | |--- max_topo >  28.00
| | | | | | | |--- class: 4
| | | | | |--- d >  0.71
| | | | | | |--- class: 1
| | | | |--- max_topo >  69.50
| | | | | |--- topo_d <= 0.29
| | | | | | |--- d <= 0.49
| | | | | | | |--- class: 4
| | | | | | |--- d >  0.49
| | | | | | | |--- class: 2
| | | | | |--- topo_d >  0.29
| | | | | | |--- mean_p_aMCs <= 0.00
| | | | | | | |--- class: 4
| | | | | | |--- mean_p_aMCs >  0.00
| | | | | | | |--- class: 2
| | | |--- topo_d >  0.48
| | | | |--- mean_p_aMCs <= 0.00
| | | | | |--- topo_d <= 0.61
| | | | | | |--- class: 1
| | | | | |--- topo_d >  0.61
| | | | | | |--- class: 4
| | | | |--- mean_p_aMCs >  0.00
| | | | | |--- max_topo <= 38.50
| | | | | | |--- topo_d <= 0.53
| | | | | | | |--- class: 4
| | | | | | |--- topo_d >  0.53
| | | | | | | |--- class: 2
```

```
|   |   |   |   |   |--- max_topo >  38.50
|   |   |   |   |   |   |--- class: 2
|   |   |--- max_topo >  423.00
|   |   |   |--- mean_p_aMCs <= 0.16
|   |   |   |   |--- n_aMCs <= 3.50
|   |   |   |   |   |--- topo_d <= 0.18
|   |   |   |   |   |   |--- d <= 0.52
|   |   |   |   |   |   |   |--- class: 2
|   |   |   |   |   |   |--- d >  0.52
|   |   |   |   |   |   |   |--- class: 4
|   |   |   |   |   |--- topo_d >  0.18
|   |   |   |   |   |   |--- mean_p_aMCs <= 0.00
|   |   |   |   |   |   |   |--- class: 2
|   |   |   |   |   |   |--- mean_p_aMCs >  0.00
|   |   |   |   |   |   |   |--- class: 2
|   |   |   |   |--- n_aMCs >  3.50
|   |   |   |   |   |--- p_recipientMC <= 0.01
|   |   |   |   |   |   |--- class: 2
|   |   |   |   |   |--- p_recipientMC >  0.01
|   |   |   |   |   |   |--- p_recipientMC <= 0.52
|   |   |   |   |   |   |   |--- class: 4
|   |   |   |   |   |   |--- p_recipientMC >  0.52
|   |   |   |   |   |   |   |--- class: 2
|   |   |   |--- mean_p_aMCs >  0.16
|   |   |   |   |--- class: 4
```
